# Supplementary material for: A possible role for hepcidin in the detection of iron deficiency in severely anaemic HIV-infected patients in Malawi
Source: PLoS One. 2020 Feb 27;15(2):e0218694. doi: 10.1371/journal.pone.0218694 (PMC7046342; doi:10.1371/journal.pone.0218694)
Supplement: S1 Table — (DOCX) [file pone.0218694.s002.docx]

**S1 Table. Accuracy of peripheral** **blood markers in relationship to hepcidin (ng/ml))**.

| **Potential markers** | **Beta** | **P-value** |
| --- | --- | --- |
| MCV (fl)^1^ | 0.02 | 0.851 |
| MCH (pg/cells) ^1^ | 0.002 | 0.975 |
| Serum iron (μmol/l) ^1^ | -0.13 | 0.322 |
| Ferritin (μg/l) ^1^ | 0.2 | 0.538 |
| sTfR receptor (mg/l)^3^ | -0.62 | 0.041 |
| sTfR index ^4^ | 8.3 | 0.001 |
| sTfR Ratio ^4^ | -2.2 | 0.310 |

Abbreviations: 95%-CI: 95% confidence interval. MCV; mean cellular volume, MCH; mean corpuscular haemoglobin, sTfR: Soluble transferrin receptor, sTfR index (sTfR(mg/L) /Log ferritin(ug/L)), sTfR Ratio (sTrR(mg/L))x1000/ferritin(ug/L)).^1^[29] ^2^[11] ^3^ [22] ^4^[21, 23]. .
