# Supplementary material for: A possible role for hepcidin in the detection of iron deficiency in severely anaemic HIV-infected patients in Malawi
Source: PLoS One. 2020 Feb 27;15(2):e0218694. doi: 10.1371/journal.pone.0218694 (PMC7046342; doi:10.1371/journal.pone.0218694)
Supplement: Attachment — Submitted filename: Respons to reviewers PLOS one manuscript 2.docx [file pone.0218694.s003.docx]

To: PLOS ONE Kostas Pantopoulos, PhD Academic Editor

Subject: Rebuttal letter resubmission Manuscript ID: PONE-D-19-15824

September 2019

Dear Kostas Pantopoulos,

We thank you for considering our manuscript titled; **“Hepcidin and conventional markers to detect iron deficiency in severely anaemic HIV-infected patients in Malawi ”** for publication in your journal. We would like to highlight that we initially submitted two manuscripts back to back. The other manuscript titled; **“Severe anaemia complicating HIV in Malawi; multiple co-existing aetiologies are associated with high mortality”** (PONE-D-19-15827) has also been resubmitted. Please receive the suggested changes and answers considering the issues raised by the reviewer. In our reply we indicated the original text (with respective line and page numbers) and changes are underlined. As requested we have tracked change all changes made in the original manuscript.

At first we would like to comment on the requested update for the funding Statement and competing Interests with regard to our author Dorine Schwinkels and *Hepcidinanalysis.com.* The statement relating to the author role contributions is adjusted: Dorine W. Swinkels is an employee of Radboud UMC that offers high quality hepcidin measurements via Hepcidinanalysis.com at a fee for service basis. *'Hepcidinanalysis.com'* did not play any role in the study design, data collection. Thank you for the online change in the submission form on our behalf.

**Major comments:**

1. Particular attention should be given on analysing hepcidin association with other iron markers. Accuracy of the title should be considered, while implications of the findings for treatment of iron deficiency in this patient population should be discussed.

- We appreciated this comment and adjusted the title: A possible role for hepcidin in the detection of iron deficiency in severely anaemic HIV-infected patients in Malawi
- Implications: we added a new line 353 (page 13): Hepcidin and (standardized) identified cut-offs are highly likely to be relevant as there is a need for a reliable marker to define BM-ID and to start iron supplementation among HIV-infected patients in resource limited settings such as Malawi. Intervention studies, using hepcidin as a marker in males and females, should be performed to assess feasibility and effect of such an intervention. In the meanwhile, as the MCV is commonly provided as part of routine full blood counts this marker may be of some use in resource-limited settings.

2 &3. General. Data availability: Have the authors made all data underlying the findings in their manuscript fully available? The author’s state that raw data will be available within the manuscript or supplementary information – it’s not clear whether this has been provided yet. It would be helpful to refer to this within the manuscript. We note that you have included the phrase “data not shown” in your manuscript. Unfortunately, this does not meet our data sharing requirements.

- We regret this comment and realised that the sentence was not additive and therefore deleted it. Line 298 page 13:

*Therefore a direct comparison is challenging. ~~At last an explanation(s) can be found in levels of infection or control of the HIV disease, which in our population was not different for woman and man (data not shown)~~.*

- All data was given within the manuscript. We did not explicitly ask for medical ethical permission to publish our database online. In case individual readers are interested in using anonymised data we can and will share our database or requested data, we have added this to the results section
- Methods were adjusted line 178 page 5; Additional raw data can be requested by contacting the corresponding author.

**Ad reviewer 1:**

4. There is a good opportunity here to investigate how hepcidin associates with other iron markers including BM-ID in the context of severe HIV-associated anaemia – where infection, inflammation, low iron status and erythroid demand may co-exist. The authors could perform univariate and multivariate analyses accordingly – in my view this would enhance the interest level of the paper, and the authors should consider performing such investigations.

- We agree that investigating the associations of hepcidin in this population is of interest to better understand factors regulating hepcidin in these patients who may have competetive stimuli. We have added the requested data concerning the associations between hepcidin and iron markers. As the aim of this paper was to assess the performance of peripheral iron marker in our population and not unravelling the competitive stimuli of hepcidin metabolism, which is a paper on itself, we have restricted the analysis to a univariate linear regression analysis. The results of this linear regression analysis are presented in table S1 and displayed in the text in line 203 (page 6): Hepcidin levels are negative correlated to sTfR receptor (mg/l)^3^ ; Beta -0.62, p=0.041) and positive towards sTfR-index (sTfR(mg/L) /Log ferritin(ug/L)); Beta 8.3, p-value 0.001), table S1.

| **Potential markers** | **Beta** | **P-value** |
| --- | --- | --- |
| MCV (fl)^1^ | 0.02 | 0.851 |
| MCH (pg/cells) ^1^ | 0.002 | 0.975 |
| Serum iron (μmol/l) ^1^ | -0.13 | 0.322 |
| Ferritin (μg/l) ^1^ | 0.2 | 0.538 |
| sTfR receptor (mg/l)^3^ | -0.62 | 0.041 |
| sTfR index ^4^ | 8.3 | 0.001 |
| sTfR Ratio ^4^ | -2.2 | 0.310 |

**Table S1. Accuracy of peripheral** **blood markers in relationship to hepcidin (ng/ml))**. Abbreviations: 95%-CI: 95% confidence interval. MCV; mean cellular volume, MCH; mean corpuscular haemoglobin, sTfR: Soluble transferrin receptor, sTfR-index (sTfR (mg/L) /Log ferritin(ug/L)), sTfR Ratio (sTfR(mg/L))x1000/ferritin(ug/L)).^1^ (29) ^2^ (11) ^3^ (29) ^4^(15, 16)

- For interpretation of the data see point 20.

5. Ethics Statement: the study does involve human participants – the authors should therefore add the ethics statement where required.

- Line 158 (page 5) was adjusted. The study was performed with respect of the confidentiality and anonymity of the research participants were participating in the study voluntarily.

6. Title: The short title is actually more informative than the main title – suggest using this or similar. (Short title: Difficulties in detection of iron deficiency in severely anaemic HIV-infected patients in Malawi)

- See previous comment 1. Title has been adjusted

7. Abstract: Line 47 – although hepcidin and MCV performed best, these are still low AUC-ROC indicating poor performance – this should be made clear. (e.g. “Although hepcidin and MCV gave the highest AUC-ROC….., all markers performed poorly in identifying BM-ID” or similar). Similar in line 52.

- We appreciate this comment and rephrased Abstract: Line 49-51 and line 54 (page2);
  - Line 43-45 (page 2): Although hepcidin and MCV performed best; AUC^ROC^ of 0.593 and 0.545, all markers performed poorly in identifying BM-ID (ROC<0.6). The AUC^ROC^ of hepcidin in males was 0.767 (sensitivity 80%, specificity 78%) and in women 0.490 (sensitivity 60%, specificity 61%).
  - Line 48-51 (page 2); It remains challenging to accurately assess iron status in severely anaemic HIV-infected patients. Further investigation of the potential of hepcidin is needed, including exploration of the discrepancy in hepcidin results between males and females.

8. Abstract: Lines 52-54 – The final line of the conclusion is not fully supported by the data which finds hepcidin to perform poorly overall in identifying BM-ID, albeit slightly less poorly than the other markers (and with moderate performance in males). Furthermore, whether iron supplementation in such settings is curative is controversial (e.g. see recent publication Haider et al, Am J Trop Med Hyg, 2019). This conclusion should be softened, e.g. state that it still remains challenging to accurately assess iron status in HIV using peripheral blood markers / say further investigation of the potential of hepcidin should be performed, especially to confirm the discrepancies between males and females.

- We appreciate the comments of the reviewer and line 53-55 of the abstract has been rephrased (see previous comment 7).
- We have now cited the Haider 2019 reference as suggested by the reviewer in the discussion: Line 307 (page 13): Moreover recently (2019) it has been shown that iron supplementation in a HIV population may be related to increased mortality (45). Therefore a reliable diagnosis of iron deficiency is important, as supplementation could put the patient at risk.

9. Introduction: Line 62 – the authors could also point out the risk of iron supplements in areas of high infectious burden (e.g. Sazawal et al, Lancet, 2006; Pasricha et al, BMJ, 2018).

- We appreciate this comment and have adjusted the introduction in line 66 (page3): Secondly although iron deficiency is preventable and treatable iron supplementations has been shown to be a risk factor for infections in areas of high infectious burden (8,9).

10. Introduction: Line 77 – reference to the BRINDA work could also be useful here (e.g. Suchdev et al, AJCN, 2017)

- In the introduction we have added a statement about the effect of inflammation on the iron metabolism line 71(page 3): Previous studies therefore concluded that peripheral blood markers of iron status, such as ferritin, might not be reliable without a correction for inflammation (14,15).

11. Introduction: Line 88 – it is also essential also to discuss hepcidin suppression during erythropoietic demand or hypoxia (e.g. via erythroferrone), especially here where all patients in the study are severely anaemic. In this section, the authors could also cite other studies/contexts where hepcidin has been investigated as a possible diagnostic for iron deficiency (e.g. ref 34 (Jonker); Pasricha et al, STM, 2014; Pasricha et al, Haematologica, 2011)

- We appreciated this comment and adjusted the introduction in line 83 (page 3): Alternative, in situations where erythropoietin demand and/or hypoxia are present (as in severe anaemia), hepcidin levels are decreased. (17-19). However, hepcidin is also unregulated by inflammation. This may limit its use in settings where infections are common (20). However hepcidin was found to be a potential marker to guide iron therapy in severely anaemic children, because in this population there is diminished up regulation of hepcidin in inflammation and iron deficiency due to an increase of erythropoietin (17). It is important to explore the possible role of hepcidin as possible useful marker in guiding iron therapy in severely anaemic adults.

12. Methods: Line 115-119 – sTfR definitions. Ref 16 shows that the Ramco assay is calibrated differently to other sTfR assays. It is essential to give further detail on the choice of cutoff – the 3.6 mg/L cutoff in 16 is based on a different assay. Furthermore, it is hard to see where the 2.75 mg/L cutoff came from; similarly, the sTfR-index cutoffs are assay dependent. This should be clarified and analyses reperformed as necessary.

We appreciate and agree with the comments of the reviewer. We have this specific test to determine sTfR to be able to compare this to previous work and since it is a practical assay to perform in a non-high tech environment. As other studies have used different methods, this may have affected the comparability of our results and we have therefor tested several previously published cut-offs and use AUC-ROC curves to identify potential improved cut-offs. As it is confusing that we presented data (table 2) for more cutoffs than we described in the methods section, we have reduced the number of cut offs presented in table 2 We furthermore added the point raised by the reviewer to the discussion:

Line 218 page 13: We have used a specific test for sTfR to be able to compare this to other work and since it is a practical assay to perform in a non-high tech environment. However other study may have used other tests, which may have affected the comparability of our results. We have therefor tested several previously published cut-offs and use AUC-ROC curves to identify potential improved cut-offs.

The presented cut-offs used were based on three previous publications. Unfortunately only one was displayed in the method section whilst others were displayed in the footnote of table 2. We have added the footnotes to the methods (line 116 page 4): For sTfR we used 2.7 mg/l and 3.6 mg/l and for the sTfR-index: 1.8 and 2.2, 2.8 respectively, as no international cut-offs have been defined, these represented the most resent consensus (21-23).

13. Methods: BM-ID. Whether or not blinding was used should be described.

- Line 135 (page 4) of the method was adjusted: The reviewing pathologist was simply provided with the patient’s identification number and was not aware of the clinical condition of the patients when reviewing the bone marrow.

14. Methods/Results: Were any inflammatory markers (e.g. CRP, AGP) assessed or could samples be reanalysed to add this data? These would aid the analysis and its interpretation. Additionally, if samples were still available, measuring erythroferrone (e.g. Intrinsic ELISA) would be very interesting.

- We appreciate the comments of the reviewer and we fully agree with the reviewer. However CRP and erythroferrone values were not determined and samples to perform retesting are not available. We adjusted the limitation section in the discussion to highlight this comment: Line 337 (page 14): Other than the presence of the tested pathogens we did not determine inflammatory markers such as CRP were not available for our patients.

15. Results: Table 1 / Lines 201-203 – hepcidin results are presented in ng/mL, yet LOD in methods is given in nmol/L – the units should be synchronised.

- We are very sorry for this misunderstanding and adjusted the method line 158-1124 (page 4): Hepcidin concentrations are expressed as nanogram per millilitre (ng/mL). The lower limit of detection of this method was 0.5 ng/mL (18).
- We further adjusted this error in the title of figure 1. **Figure 1. Hepcidin (ng/mL) ROC curve by gender with optimal cut-off**

16. Results: Table 2 – hepcidin data should be added to this table for completion - it is not particularly clear linked to Figure 1 (partly due to resolution issues).

- Several adjustments have been made to clarify this point:
  - Hepcidin information was moved from figure 1 into table 2.
  - The legend for figure 1 was adjusted: **Figure 1. Hepcidin (**ng/mL**) ROC curve by gender with optimal cut-off.** The best cut-off value for diagnosing BM-ID, table 1, was determined by the Youden index (maximum (sensitivity + specificity −1)) in the ROC-curve (25). Hepcidin overall; AUC^ROC^ 0.593, optimal cut-off ≤7 ng/mL (sensitivity 67% & specificity 67%). Hepcidin in man; AUC^ROC^ 0.767 optimal cut-of ≤6 ng/mL (sensitivity 80%; specificity 78%). Hepcidin in women 0.490 optimal cut-off ≤7 ng/ml (sensitivity 60% & specificity 61%. Abbreviations: AUC^ROC^: Area Under Curve of Receiver Operating Characteristic.

17. Discussion – line 243/244 – see point above on abstract-line 47. Suggest changing the emphasis to indicate that they are still generally poor performing tests in this setting – this raises the question of its suitability for use in this setting.

- We agree with the reviewer and adjusted line 246 (page 12): In the first study evaluating hepcidin as a marker for BM-ID among severely anaemic HIV-infected adults in this setting we found all tested markers performed suboptimal in detecting BM-ID. Hepcidin was the best performing marker but also had a suboptimal accuracy (AUC^ROC^ 0.593), which was less pronounced in males (AUC^ROC^ 0.767). MCV was found to be the best, suboptimal conventional peripheral blood marker for BM-ID. As the MCV is commonly provided as part of routine full blood counts this marker may be of some use in resource-limited settings.

18. Discussion – line 249-250: the authors should add supporting evidence from the literature that ART associates with iron-deficiency (specifically, not just anaemia). As far as we know only ART including Protease Inhibitors (PI) can be associated with iron deficiency directly. As in our study cohorts PI’s were not used we prefer not to make a comment on this topic in the current manuscript. Reference: [Yusuf E. Afacan](https://www.ncbi.nlm.nih.gov/pubmed/?term=Afacan%20YE%5BAuthor%5D&cauthor=true&cauthor_uid=11853049) [Yusuf E. Afacan](https://www.ncbi.nlm.nih.gov/pubmed/?term=Afacan%20YE%5BAuthor%5D&cauthor=true&cauthor_uid=11853049), [Muhammad S. Hasan](https://www.ncbi.nlm.nih.gov/pubmed/?term=Hasan%20MS%5BAuthor%5D&cauthor=true&cauthor_uid=11853049), and  [Jackson A. Omene](https://www.ncbi.nlm.nih.gov/pubmed/?term=Omene%20JA%5BAuthor%5D&cauthor=true&cauthor_uid=11853049) **Iron deficiency anemia in HIV infection: immunologic and virologic response.** [J Natl Med Assoc](https://www.ncbi.nlm.nih.gov/pmc/articles/PMC2594130/). 2002 Feb; 94(2): 73–77.

19. Discussion - line 264, 270, 308-311: attention is drawn to the recently published large study on anaemia and mortality in HAART-treated HIV in which iron supplements during HAART were associated with increased mortality (Haider et al, Am J Trop Med Hyg, 2019). It would be useful to draw this into the discussion.

- We agree with the reviewer and changed the discussion to highlight the results published by Haider et al; see comment 8.

20. Discussion – line 273: besides inflammation, the potential involvement of hepcidin suppressive signals during severe anaemia should also be considered (e.g. is there a negative association between hepcidin and sTfR concentrations).

- We appreciate the comment of the reviewer. As raised earlier we have evaluated the association between hepcidin and sTfR concentration with a linear regression model, table 3. See comment 4. The coefficient was indeed negative (-0.11) and reached significance. This may be explained by the fact that inflammation is an important factor upregulating hepcidin, whilst does not affects sTfR levels. Without markers to test for inflammation this remains speculative though. We have included this in the discussion: – Line 340 (page 14): Hepcidin may be influenced by multiple conflicting stimuli in this patient population including, iron deficiency, anaemia/hypoxia and inflammation. We found an inverse association between hepcidin and sTfR, which may indicate that inflammation was an important stimulus of hepcidin levels in our population. Without in-depth analysis including all relevant stimuli and repeated sampling it is very difficult to understand the complexity of hepcidin and iron metabolism in our population. Low hepcidin values could indicate that iron supplementation would be indicated and effective in reducing anaemia caused by iron deficiency. However the effectiveness and safety should be evaluated in a prospective trial design in this population.

21. Formatting and spelling

- Formatting / Spelling: Authors: “Dorine Swinkels” (not “Dorien”): This has been corrected
- Formatting / Spelling: Methods – Statistics: Line 165 – sentence is unclear (“confounding was enhanced…”): This was rephrased line 165 (page 5): To assess any confounding factors, hepcidin concentrations were evaluated by gender, HIV disease progression, the use of ART as baseline, and TB infection (Pearson Chi-square test).
- Formatting / Spelling: Line 299: “men” and “women”. This has been corrected.
- Formatting / Spelling: Reference 37: “Reference values…” This has been corrected.

**Reviewer 2**

**Major comments:**
22. Hepcidin as a serological test to evaluate for iron deficiency is relatively expensive and not readily available compared to other more conventional tests such as complete blood counts (MCV, MCH), ferritin levels, serum iron and even soluble transferrin receptor. Given that hepcidin only marginally performed better than MCV in the AUCROC analysis (0.59 vs 0.55, both low diagnostic accuracy at <0.7), will the authors strongly recommend this as the best possible peripheral test for evaluation of iron deficiency in HIV infected populations in resource limited settings with high levels of inflammation?

- We fully agree that none of the tested markers were optimal; including hepcidin and that given the current costs hepcidin is of little added value. Since Hepcidin may be predictive of iron supplementation success and safety we believe our results are important. Until this has been proven and when only conventional markers are available, MCV could still be used, We have now clarified this in the text and have also highlighted (with a new reference - Haider 2019), see comment 8 & 19, The importance of avoiding unnecessary iron supplementation in HIV-infected adults.

23. It is interesting that the prevalence of BM iron deficiency is relatively high in this study population of HIV infected, severely anaemic adults- 48%, similar to the non-HIV infected population and prevention and treatment of iron deficiency still plays a vital role in management of severely anaemic HIV infected patients. Were there other potentially treatable causes of severe anaemia noted by the authors? for example, from the baseline table, the percentage of the study population with high viral loads (>1000 copies/ml) was very high at 75% and this could have also contributed significantly to anaemia in this population.

- We fully agree with the reviewer’s comment. We have submitted a ‘back-to-back’ manuscript: **“Severe anaemia complicating HIV in Malawi; multiple co-existing aetiologies are associated with high mortality”** in which we describe the relationship between different anaemia aetiologies in the same patient population and have therefore not repeated these findings in this paper.

24. Iron supplementation has been found to increase the risk of malaria and other infectious diseases in Malawian HIV infected children from previous randomized controlled trials. Given that iron deficiency strongly contributes to the aetiology of anaemia in this study population of HIV infected adults, how do the authors suggest that this problem (found in nearly half of your study population) be addressed?

- We agree that iron supplementation is not without risk so we do not suggest that all anaemic patients with HIV should start iron supplementations. However, we suggest that there might be a role for hepcidin in the future to guide clinicians about the need for iron supplements. We have adjusted the text to take account of this. See comment 8 &19.

25. stfr receptor had an AUC of 0.52 (low diagnostic accuracy) but a better sensitivity (71%) than hepcidin (67%) and (MCV 42%) from the findings of this study. would this not be a better screening tool for iron deficiency than hepcidin given the availability and cost? or are the authors going strictly by the AUC, which for both markers (hepcidin and stfr receptor) are equally poor?

- We agree with the reviewer that we may not have highlighted this clearly in our discussion. We have now added the information: line 316 page 13: Although sTfR had a poor AUC^ROC^ it did have a better sensitivity than e.g. hepcidin and thus may be used to screen for iron deficiency

**Minor comments:**
26. Why are p values provided in table 1? Are the authors trying to tell us that the iron markers, infection rates, HIV tests and nutritional status did not differ significantly in the BM-ID vs non BM-ID group?

- In the method line 195 we state: “ Baseline characteristics were compared between BM-ID and non-deficient patients using Chi-square test (dichotomous data) or t-test (continuous) or Pearson Chi-square test (continuous not normally distributed).” As the reviewer has noted, we did this to provide clear data on BM-ID and to understand the relationship between potential confounding factors in the clinical outcome. However we agree this information is not very useful to the reader. We have there for deleted the column and added a footnote: all tested p-values were > 0.1.

27. Could the authors offer further explanation as to why the hepcidin test performed better in males than females, with the test over 0.7 in males with sensitivity of 80% and sensitivity of 78% compared with females, with the AUC of <0.5 with poorer sensitivity and specificity?

As we described in the discussion, we do not have a good explanation why hepcidin performed better in men as compared to women. Age and gender differences in hepcidin concentrations and thus cut offs to detect ID have been repeatedly reported. As described in the discussion(line 303; page 13), these reference data have detected in European subjects and currently no hepcidin reference levels have been published for an African population. Line 303 (page 13) was rephrased: Therefore a direct comparison is challenging and a clear clarification of the gender differences difficult.

28. Discussion, line 272, second sentence: 'it is not surprising that hepcidin remains from perfect as a marker for BM-ID” should be changed to ‘it is not surprising that hepcidin remains far from perfect as a marker

- Adjusted as requested.

We hope to have clarified outstanding questions and improved the manuscript according to the concerns raised. Please do not hesitate to contact us if you have any further questions.

With many thanks for your consideration, and on behalf of all the authors.

Minke Huibers, MD
